# *Mdm1* ablation results in retinal degeneration by specific intraflagellar transport defects of photoreceptor cells

**DOI:** 10.1038/s41419-022-05237-2

**Published:** 2022-09-28

**Authors:** Youlim Son, Soo-Jin Kim, Hwa-Young Kim, Junyeop Lee, Jae-Ryong Kim

**Affiliations:** 1 Department of Biochemistry and Molecular Biology, Yeungnam University, Daegu, 42415 Republic of Korea; 2 Smart-aging Convergence Research Center, College of Medicine, Yeungnam University, Daegu, 42415 Republic of Korea; 3 Department of Ophthalmology, Asan Medical Center, University of Ulsan, College of Medicine, Seoul, 05505 Republic of Korea

**Keywords:** Retina, Visual system

## Abstract

Mouse double minute 1 (*Mdm1*) might be involved in the function and structure of centrioles and age-related retinal degeneration. However, the mechanism by which Mdm1 deficiency causes retinal degeneration remains unknown. We confirmed that the Mdm1 protein is localized at the connecting cilium (CC) of photoreceptor cells in the retina. The electroretinograms of 6-week-old *Mdm1*^−/−^ mice revealed decreased vision, which was eventually lost, and outer segment (OS) photoreceptor degeneration was evident on postnatal day 7, with complete loss of the outer nuclear layer (ONL) observed at 35 weeks. *Mdm1*^−/−^ mouse retinas showed mislocalization of opsins in the photoreceptor cells, indicating particular intraflagellar transport (IFT) defects, and entrapment of the nuclei in the ONL by microvilli of retinal pigment epithelial cells, leading to apoptosis in the ONL. These results suggest that *Mdm1* ablation causes specific IFT defects, which prevents the OS from continuously replenishing new discs, resulting in retinal degeneration.

## Introduction

The mouse double minute (*Mdm*) genes *Mdm1* and *Mdm2* were initially found in amplified extrachromosomal DNA sequences termed double minutes in a spontaneously transformed derivative of mouse 3T3 cells [1]. *Mdm2*, an E3 ubiquitin ligase, binds and induces p53 degradation, exhibiting oncogenic activity as a negative regulator of the p53 tumor suppressor [2]. In contrast, *Mdm1* shows no tumorigenic activity [3] and has been identified in the centrosome in cells [4]. Mdm1 was upregulated during ciliogenesis in multiciliated tracheal epithelial cells and localized to centrioles and cilia when transfected into NIH/3T3 cells [5]. Mdm1 is a microtubule-binding protein that inhibits centrosome duplication when overexpressed [6]. Proteomic analysis revealed that Mdm1 was also detected in the mouse photoreceptor sensory cilium complex and in the outer segment (OS) of photoreceptor cells in the retina [7], suggesting that *Mdm1* might be involved in the regulation of the functions and structure of the centrosome, as well as cilia.

A nonsense mutation in the *Mdm1* gene was identified in a naturally occurring mouse strain that developed age-related retinal degeneration (arrd2) with retinal pigment epithelium (RPE) atrophy, vessel attenuation, pigment abnormalities, and complete loss of photoreceptor cells with age [8], suggesting that *Mdm1* might play an important role in the function and structure of photoreceptor cells of the retina in mice.

Cilia are present on the surface of most eukaryotic cells and contain an axoneme, which begins at the basal bodies and passes through a transition zone [9]. Ciliogenesis and maintenance of cilia require intraflagellar transport (IFT), which is a process mediated by molecular motors and IFT particles. During IFT, nonmembrane-bound particles are moved continuously along the axonemal doublet microtubules from the base to the tip of a cilium (anterograde) or from the tip of the cilium to the base of the cilium (retrograde) [10]. Mutations in IFT genes inhibit the assembly and maintenance of cilia [11]. The photosensitive OS of cone and rod photoreceptor cells is a specialized counterpart of the primary cilium that encompasses protein complexes that transport proteins and lipids. Thus, similar to primary cilium machinery, IFT machinery is important for the development and maintenance of photoreceptor cells [12]. Therefore, defects in ciliary function and IFT are associated with retinal degeneration or dystrophy in mice and humans [13].

Although some evidence suggests that Mdm1 might play an important role in the function and structure of cilia and may be associated with age-related retinal degeneration in mice, the mechanism by which Mdm1 deficiency results in retinal degeneration remains unknown. Hence, in the present study, we used the CRISPR/Cas9 system to generate *Mdm1*^−/−^ mice and investigated the role of Mdm1 in retinal degeneration. We demonstrated that Mdm1 is localized at the axoneme of cilia in cells of various tissues, notably at the connecting cilium (CC) of photoreceptor cells in the retina. We characterized retinal degeneration in *Mdm1*^−/−^ mice and determined that Mdm1 deficiency causes defects in certain IFT, thereby leading to retinal OS disruption and degeneration.

## Results

### Generation of *Mdm1*-knockout mice by CRISPR/Cas9 technology

**Fig. 1:**
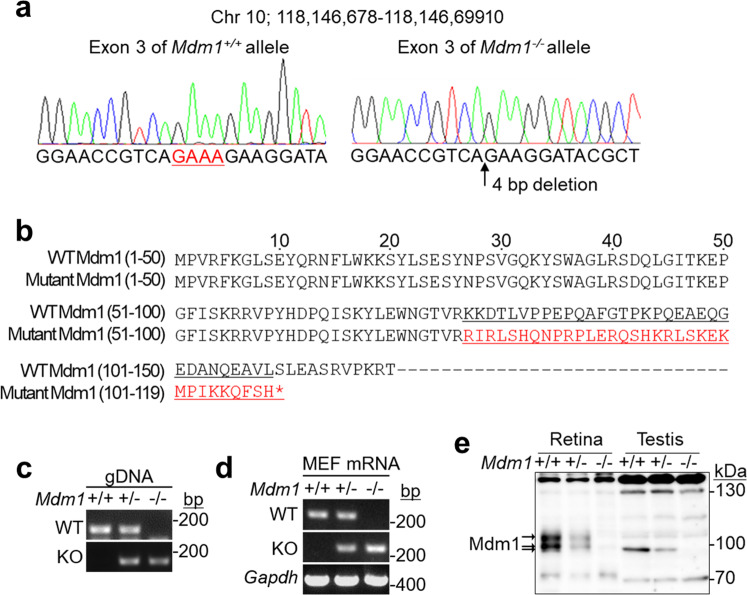
Generation of *Mdm1*^−/−^ mice using a CRISPR/Cas9 system. **a** The DNA sequences of *Mdm1*^+/+^ and *Mdm1*^−/−^ alleles in *Mdm1* exon 3. The deletion of 4 bp was confirmed in the *Mdm1*^−/−^ alleles. **b** The putative amino acid sequences of wild-type and mutant Mdm1 proteins. **c** The genotyping of *Mdm1*^+/+^, *Mdm1*^+/−^, and *Mdm1*^−/−^ mice. **d** The RT-PCR analysis of *Mdm1* expression in *Mdm1*^+/+^, *Mdm1*^+/−^, and *Mdm1*^−/−^ MEFs. *Gapdh* expression was used as a loading control for cDNA. **e** The expression levels of Mdm1 protein in the retinas and the testes of *Mdm1*^+/+^, *Mdm1*^+/−^, and *Mdm1*^−/−^ mice as confirmed by Western blotting. gDNA, genomic DNA; MEFs, mouse embryonic fibroblasts; WT, wild-type; KO, knockout.

*Mdm1*-knockout mice were generated from C57BL/6N mice using the CRISPR/Cas9 system with sgRNAs targeting exon 3 containing the first codon. The deletion of 4 base pairs (bp) in *Mdm1* exon 3 was confirmed by DNA sequencing analysis using primers spanning the target sites (Fig. 1a). In *Mdm1* exon 3, the deletion of these 4 bp induced a frameshift mutation, thereby resulting in a truncated form of the Mdm1 protein (Fig. 1b). This mutant Mdm1 protein consisted of 109 amino acids, of which 76 N-terminal amino acids are identical to a sequence in the wild-type (WT) Mdm1 protein consisting of 708 amino acids (Fig. 1b). The *Mdm1* genotypes in mice were confirmed by PCR genotyping (Fig. 1c). To analyze *Mdm1* mRNA transcripts, RNAs were extracted from MEFs of *Mdm*^*+/+*^, *Mdm*^*+/−*^*,* or *Mdm1*^*−/−*^ mice, after which RT-PCR was performed using primers that bind to *Mdm*^*+/+*^ or *Mdm1*^*−/−*^ transcripts. The RT-PCR analysis revealed a WT mRNA transcript in *Mdm1*^+/+^ and *Mdm1*^+/−^ MEFs and a knockout (KO) transcript in *Mdm1*^+/−^ and *Mdm1*^−/−^ MEFs (Fig. 1d). Western blot analysis revealed that the Mdm1 proteins were detectable as 97 kDa and 102 kDa in the retina and as 95 kDa in the testis of *Mdm1*^+/+^ mice (Fig. 1e), which is consistent with a previous report that there are two retinal-specific *Mdm1* transcripts in the retina [8]. In contrast, these Mdm1 proteins were not detectable in the *Mdm1*^−/−^ mice (Fig. 1e). These results indicate that the *Mdm1*^−/−^ mice harbor a 4 bp deletion mutation in *Mdm1* exon 3, leading to a frameshift in the *Mdm1* gene and possibly resulting in a truncated form of the Mdm1 protein.

### Localization of the Mdm1 protein in ciliated cells of various tissues in mice

**Fig. 2:**
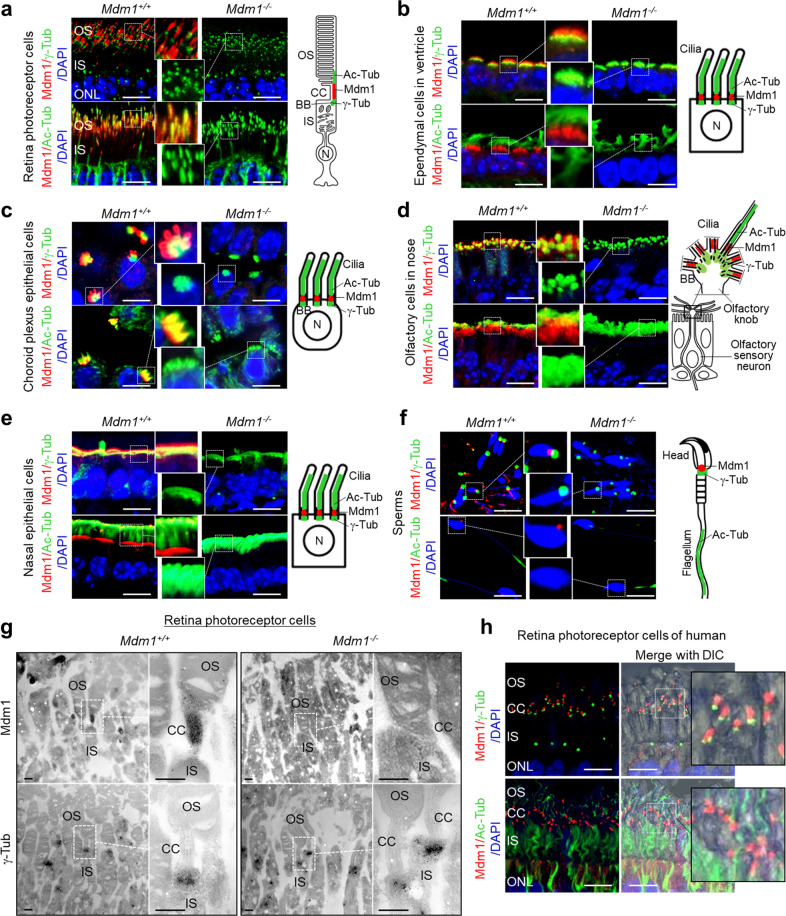
Localization of Mdm1 protein in the cilia of various tissues in *Mdm1*^+/+^ and *Mdm1*^−/−^ mice. Formalin-fixed paraffin-embedded tissue sections from *Mdm1*^+/+^ and *Mdm1*^−/−^ mice were stained with antibodies against Mdm1 (red), γ-tubulin (green), or acetylated-tubulin (green) and then counterstained with DAPI (blue) to visualize the nuclei. Representative images are shown (*n* > 8 per group). The expression of each protein was analyzed by confocal microscopy and the localization of each is shown with schematic cartoons (**a**–**f**). **a** Photoreceptor cells of the retina. **b** Ependymal cells of the ventricle. **c** Choroid plexus epithelial cells. **d** Olfactory cells in the nose. **e** Nasal epithelial cells. **f** Sperm in the testis. Scale bar, 10 μm. **g** Immunoelectron microscopy was performed to determine the localization of the Mdm1 protein in photoreceptor cells of the retina. Scale bar, 500 nm. **h** Immunofluorescence staining of Mdm1 (red), γ-tubulin (green) or acetylated-tubulin (green) in the human retina. Scale bar, 10 μm. OS, outer segment; IS, inner segment; CC, connecting cilium; BB, basal body; ONL, outer nuclear layer.

Since the Mdm1 protein localizes to centrioles and cilia in cells [5], its location was analyzed in cultured cells and various ciliated cells from mice by immunofluorescence staining. γ-Tubulin, a centrosomal marker, and acetylated α-tubulin, a marker of the ciliary axoneme, were used to verify the localization of the Mdm1 protein in ciliary cells. Mdm1 costained with γ-tubulin, confirming that it was located at the centrosome in human retinal pigmented epithelial cells (hRPE) and in *Mdm*^*+/+*^ MEFs, but no Mdm1 was detected in *Mdm*^*−/−*^ MEFs (Supplementary Fig. S1a). In the photoreceptor cells of the mouse retina, stained γ-tubulin was observed at the centrioles of the basal body complex, and acetylated α-tubulin was observed at the ciliary axoneme that stabilizes the OS of photoreceptor cells in the retina (Fig. 2a). Mdm1 staining was mainly observed in the CC and partially colocalized with acetylated α-tubulin in photoreceptor cells (Fig. 2a). Stained Mdm1 protein was observed between the basal body complex and apical cilia of cuboidal epithelial cells among the ependymal cells of the cerebral ventricle (Fig. 2b); in the choroid plexus, which is a modified ependyma (Fig. 2c); the olfactory cilia in the olfactory knob at the end of olfactory sensory neurons (Fig. 2d); and the respiratory epithelium in the nose (Fig. 2e). In testicular sperm, Mdm1 has been found between the nucleus and the centriole in the sperm neck (Fig. 2f). No staining of Mdm1 protein was detected in the examined tissues of *Mdm*^−/−^ mice (Fig. 2a–f). To further confirm the localization of the Mdm1 protein, immunoelectron microscopic analysis was performed. As expected, in the photoreceptor cells of the retina, the Mdm1 protein localized at the CC connecting the inner segment (IS) and OS and was not in the centriole of the basal body complex with stained γ-tubulin (Fig. 2g). In other ciliated cells, the Mdm1 protein was found to be more frequently localized at the more apical side than at the centriole of the basal body complex (Supplementary Fig. S1b–e). No Mdm1 staining was observed in the examined tissues of *Mdm1*^−/−^ mice (Fig. 2g and Supplementary Fig. S1b–e). In addition, we examined the localization of the Mdm1 protein in the human retina. The Mdm1 protein was localized at the CC of photoreceptor cells (Fig. 2h). Taken together, these observations revealed that the Mdm1 protein is located between the centriole of the basal body complex and ciliary axoneme in ciliated cells and between the nucleus and centriole in sperm.

### Retinal examination of *Mdm1*^+/+^ and *Mdm1*^−/−^ mice

**Fig. 3:**
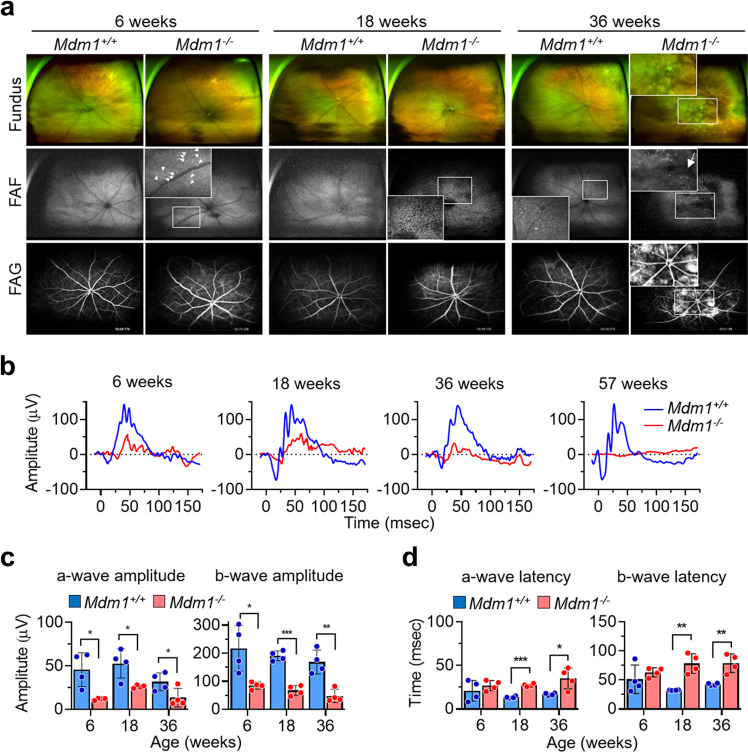
Retinal phenotypes of *Mdm1*^+/+^ and *Mdm1*^−/−^ mice. **a** Representative fundus photographs, fundus autofluorescence (FAF), and retinal fluorescein angiograms (FAG) of the retinas of *Mdm1*^+/+^ or *Mdm1*^−/−^ mice at the indicated ages. **b** Representative combined rod and cone electroretinograms. **c** Quantification of a-wave and b-wave amplitudes. **d** Quantification of a-wave and b-wave latencies. Values are the means ± SEM; *n* = 4 per group (*P < 0.05, ** *P* < 0.01, *** *P* < 0.001 by a two-tailed Student’s *t*-test).

Since age-related retinal degeneration (arrd2) in a mouse strain has previously been reported to be due to a nonsense mutation in the *Mdm1* gene [8], we examined retinal degeneration in *Mdm1*^+/+^ and *Mdm1*^−/−^ mice. *Mdm1*^+/+^ mice showed normal fundus and blood vessel structures, as judged by fundus examination, near-infrared fundus autofluorescence (FAF), and fluorescein angiography (FAG) (Fig. 3a). However, 36-week-old *Mdm1*^+/+^ mice showed some white speckles with hyperautofluorescence, representing increased melanolipofuscin derived from photoreceptor degeneration in FAF (Fig. 3a). In contrast, *Mdm1*^−/−^ mice had white speckles (Fig. 3a, arrowheads), which appeared at 6 weeks of age and became more prominent with advancing age (Fig. 3a). FAG of 36-week-old *Mdm1*^−/−^ mice revealed hyperautofluorescent spots that matched with the fundus photographs, indicating photoreceptor degeneration, and hypopigmented patchy lesions with hypoautofluorescence as determined by FAF, indicating RPE loss (Fig. 3a, arrow). Visual function was assessed by electroretinography (ERG). ERG responses in *Mdm1*^−/−^ mice diminished over time, while the responses in *Mdm1*^+/+^ mice remained stable. The difference in electroretinograms between *Mdm1*^+/+^ and *Mdm1*^−/−^ mice was notable at 6 weeks of age (Fig. 3b), and both a-wave and b-wave amplitudes were significantly reduced in the *Mdm1*^−/−^ mice compared to those in the *Mdm1*^+/+^ mice (Fig. 3c). The latency of the a-wave and b-wave also increased in the *Mdm1*^−/−^ mice with age (Fig. 3d). These findings demonstrated that Mdm1 deficiency induces early-onset photoreceptor degeneration, which continues to progress into profound RPE loss with age, resulting in visual dysfunction in mice.

### Histological and electron microscopy analyses of the retinas in *Mdm1*^+/+^ and *Mdm1*^−/−^ mice

**Fig. 4:**
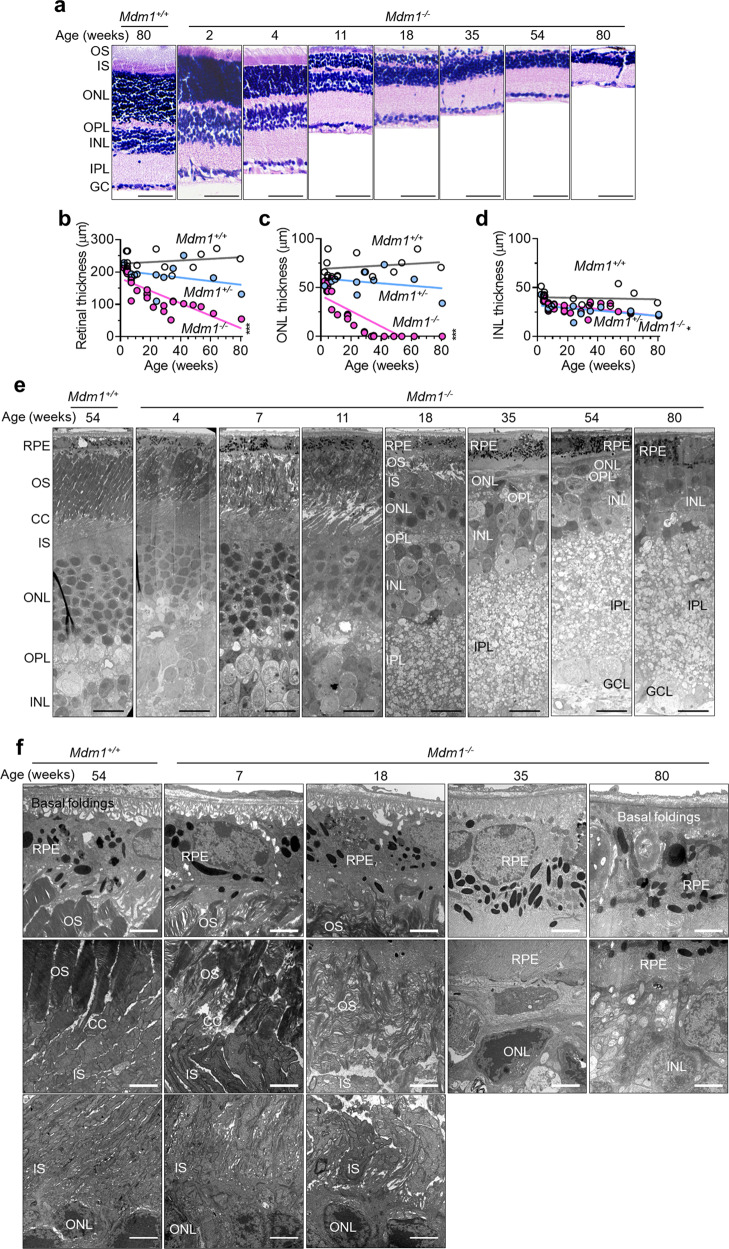
Histological and TEM analyses of retinal structures in *Mdm1*^+/+^ and *Mdm1*^−/−^ mice. **a** Representative images of hematoxylin and eosin (H&E) stained retinal sections of *Mdm1*^+/+^ or *Mdm1*^−/−^ mice at the indicated ages. Scale bar, 50 μm. Quantification of retinal thickness of *Mdm1*^+/+^ and *Mdm1*^−/−^ mice with increasing age (**b**–**d**) (*n* = 15, 10, and 27 mice in *Mdm1*^+/+^, *Mdm1*^+/−^, and *Mdm1*^−/−^ mice, respectively). **b** Total retinal thickness. **c** ONL thickness. **d** INL thickness. **e** TEM images of the retinas in female *Mdm1*^+/+^ or *Mdm1*^−/−^ mice of the indicated ages. Scale bar, 10 μm. **f** Ultrastructural analysis of the retinas in *Mdm1*^+/+^ or *Mdm1*^−/−^ mice at the indicated ages. Scale bar, 2 μm. GC, ganglion cell; IPL, inner plexiform layer; INL, inner nuclear layer; OPL, outer plexiform layer; ONL, outer nuclear layer; IS, inner segment; OS, outer segment; RPE, retinal pigment epithelium; CC, connecting cilium. Statistical analysis, linear regression.

To confirm retinal degeneration in mice, the retinal structures of *Mdm1*^+/+^ and *Mdm1*^−/−^ mice were evaluated histologically. The cellular layers and thickness of the retina were well maintained in 80-week-old *Mdm1*^+/+^ mice (Fig. 4a). In contrast, *Mdm1*^−/−^ mice showed gradual reduction in the OS, IS, and ONL of photoreceptor cells in the retina, resulting in decreased retinal thickness starting at 4 weeks and continuing with age (Fig. 4a). Complete loss of the ONL was observed in 35-week-old *Mdm1*^−/−^ mice. Total retinal thickness and ONL thickness was significantly decreased with age in the *Mdm1*^−/−^ mice (Fig. 4b, c). INL thickness also decreased slightly in the *Mdm1*^−/−^ mice (Fig. 4d). *Mdm*^*+/−*^ mice showed no retinal degeneration, as confirmed by retinal thickness and ONL thickness measures (Fig. 4b–d) and by histological examination (data not shown). These results suggest that *Mdm1* ablation causes retinal degeneration with the loss of OS, IS, ONL, OPL and some INL in mice.

TEM analysis was performed to investigate the ultrastructural changes of the retina. In the 54-week-old *Mdm1*^+/+^ mice, a highly ordered multilayered retina was observed (Fig. 4e). A single sheet of cuboidal retinal pigment epithelium (RPE) had basal folding in the choroid direction and microvilli, which were interdigitated with the OS of photoreceptor cells. The OS of photoreceptor cells had organized transverse stacks of membranous discs and was connected to the IS by CC (Fig. 4f). In contrast, *Mdm1*^−/−^ mice showed the gradual loss of OS, IS, and ONL in the retina with age (Fig. 4e, f). The rate of progressive retinal degeneration with age was similar between male and female *Mdm1*^−/−^ mice (Supplementary Fig. S2a), suggesting no sexual difference in retinal degeneration in *Mdm1*^−/−^ mice. In the retinas of 7-week-old *Mdm1*^−/−^ mice, disruption of the OS and CC architecture and longitudinal fragmentation of the IS were observed, and these changes were more profound at 18 weeks (Fig. 4f and Supplementary Fig. S2b). As the disruption of OS and IS progressed, multilayered autophagic vesicles were observed in 7-week-old *Mdm1*^−/−^ mice (Supplementary Fig. S2c, arrows). In 35-week-old *Mdm1*^−/−^ mice, the retina exhibited disrupted basal folding of the RPE, complete loss of OS and IS, loss of most ONL nuclei, and entrapment of the remaining ONL nuclei by RPE processes (Fig. 4e, f). In 80-week-old *Mdm1*^−/−^ mouse retinas, complete loss of the ONL and outer plexiform layer (OPL) was evident, and RPE processes invaded the inner nuclear layer (INL) and dislocated the remaining nuclei (Fig. 4e, f).

Since retinal degeneration was reported to be observed at 6 months of age in *arrd2* mice [8] and because we observed that *Mdm1*^−/−^ mice showed retinal degeneration as early as the 7th week of age, we tried to identify a time point at which OS degeneration begins in *Mdm1*^−/−^ mouse retinas. At postnatal day 5 and 7 (P5 and P7), OS had not yet developed in the mouse retina, and therefore, no OS degeneration was evident in either the *Mdm1*^+/+^ or *Mdm1*^−/−^ mice (Supplementary Fig. S2d). Because mice open their eyes 9–14 days after birth [14], we also investigated the effects of light exposure on OS degeneration and development in the retinas of P10 mice with eyes open compared to P10 mice with closed eyes. Light exposure accelerated OS development in the *Mdm1*^+/+^ mice, whereas it facilitated OS degeneration in the *Mdm1*^−/−^ mice (Supplementary Fig. S2e). These results suggest that OS degeneration due to *Mdm1* deficiency is observed from the 10th day, and light exposure further promotes OS degeneration in the *Mdm1*^−/−^ mice.

### Involvement of Mdm1 in specific intraflagellar transport (IFT)

We investigated the mechanism by which *Mdm1* deficiency induces retinal degeneration, which is initiated by OS disruption. Since immunofluorescence staining and immunoelectron microscopy revealed that the Mdm1 protein was localized to the CC of photoreceptor cells, we first examined whether the structure of the CC was altered or collapsed with age. Although disruption of OS and IS progresses with age, the structure of the CC was well organized at 7 weeks and maintained at 22 weeks (Supplementary Fig. S3a). These results suggest that it is unlikely that *Mdm1* deletion primarily causes deformation of the CC structure, and therefore, it is unlikely to cause OS disruption or retinal degeneration.

**Fig. 5:**
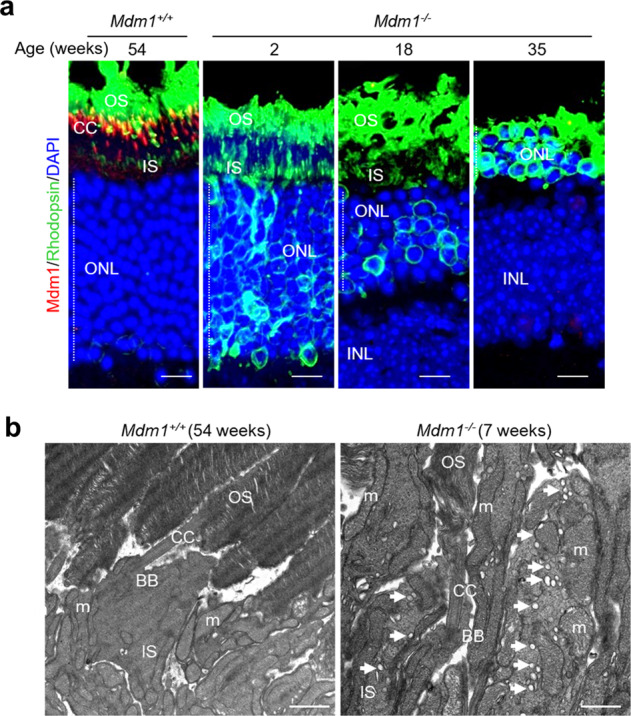
Distribution of rhodopsin and vesicles in the photoreceptor cells of the retinas of *Mdm1*^+/+^ and *Mdm1*^−/−^ mice. **a** Immunofluorescence staining of Mdm1 (red) and rhodopsin (green) in the photoreceptor cells of *Mdm1*^+/+^ and *Mdm1*^−/−^ mice at the indicated ages. Representative images are shown (*n* > 5 per group). Scale bar, 10 μm. **b** TEM images of the photoreceptor cells of the retina in *Mdm1*^+/+^ and *Mdm1*^−/−^ mice. Arrows indicate vesicles in the IS. Scale bar, 1 μm. RPE, retinal pigmented epithelial cells; OS, outer segment; CC, connecting cilium; BB, basal body; IS, inner segment; ONL, outer nuclear layer; INL, inner nuclear layer; m, mitochondria.

Since the Mdm1 protein was localized at the CC of photoreceptor cells, we speculated that Mdm1 might be involved in IFT, which is important for photoreceptor development and maintenance [12]. Therefore, we used immunofluorescence staining to measure the distribution of rhodopsin, which is the most abundant protein in rod photoreceptor cells in the retina and is transported from the IS to the disc of OS through the CC via IFT [15]. Rhodopsin was localized in the OS and not observed in the ONL in the *Mdm1*^+/+^ mice (Fig. 5a). In contrast, rhodopsin was distributed not only in the OS but also in the IS and ONL in the *Mdm1*^−/−^ mice at 2 weeks of age and remained in the ONL at 35 weeks (Fig. 5a). In addition to rhodopsin, we observed the distribution of S-opsin and M/L-opsin involved in visual signal transduction in cone photoreceptors. Both S-opsin and M/L-opsin, like rhodopsin, accumulated in the IS and ONL of *Mdm1*^−/−^ mice, suggesting that opsins are mislocalized due to defects in IFT (Supplementary Fig. S3b). We examined the localization of another phototransduction proteins, cone-specific transducin alpha subunit (GNAT2) and cone arrestin. There was no difference in the distribution of GNAT2 and arrestin in retinas between 7-week-old *Mdm1*^*+/+*^ and *Mdm1*^−/−^ mice (Supplementary Fig. S3c). Electron microscopy also revealed the accumulation of vesicles in the IS of photoreceptor cells in the *Mdm1*^−/−^ mice, which was not observed in the *Mdm1*^+/+^ mice (Fig. 5b, arrows). These results suggest that *Mdm1* deficiency causes defects in specific IFT, leading to mislocation of opsins and preventing the OS from continuously replenishing new discs, thereby leading to OS disruption.

### Apoptosis was observed in the ONL of *Mdm1*^*−/−*^ mice

**Fig. 6:**
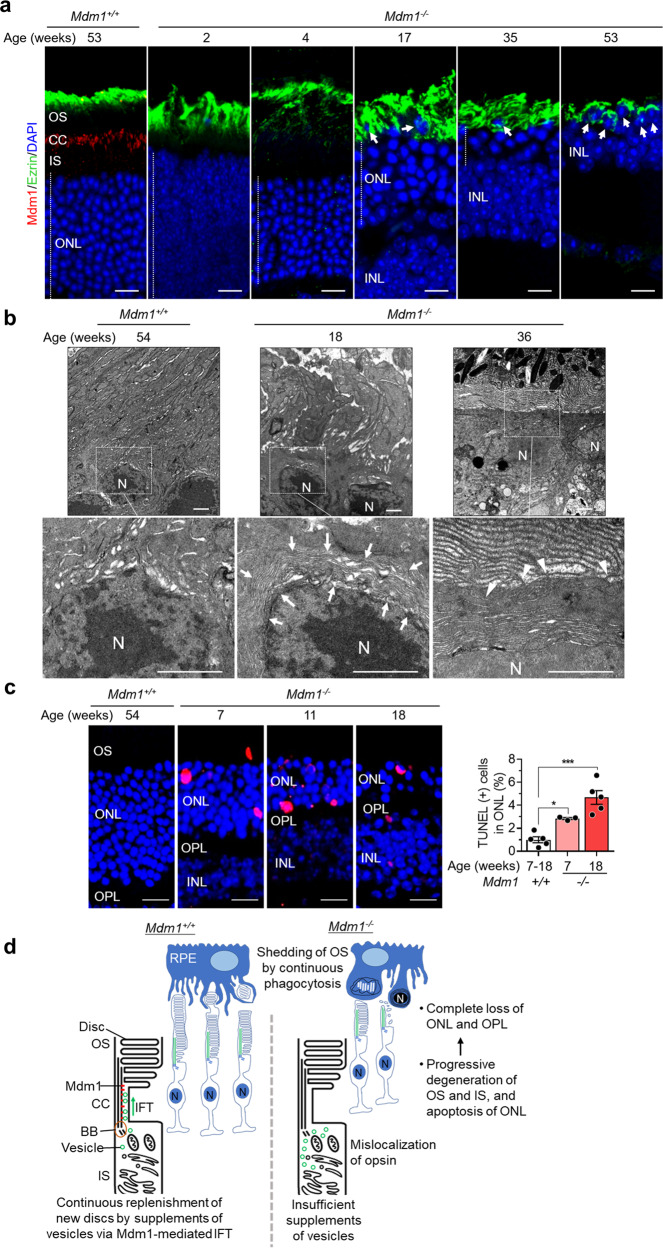
Distribution of RPE microvilli and apoptosis of the nuclei in the ONL of *Mdm1*^+/+^ and *Mdm1*^−/−^ mice. **a** Immunofluorescence staining of Mdm1 (red) and Ezrin (green) in the retinas of *Mdm1*^+/+^ or *Mdm1*^−/−^ mice at the indicated ages. Representative images are shown (*n* > 5 per group). Arrows indicate the nucleus surrounded by RPE microvilli. Scale bar, 10 μm. **b** TEM images of the ONL of the retina in *Mdm1*^+/+^ and *Mdm1*^−/−^ mice. Arrows indicate the RPE microvilli surrounding the nucleus. Arrowheads indicate microvilli extending from the RPE. Scale bar, 1 μm. N, nucleus. **c** TUNEL staining and quantification of TUNEL-positive cells of the ONL in the retinas of *Mdm1*^+/+^ or *Mdm1*^−/−^ mice. The TUNEL staining photograph shows a representative image obtained from the center of the retina. The quantitative graph shows the percentage of TUNEL ( + ) cells in the total ONL cells including both the central and peripheral parts of the retina. Scale bar, 10 μm. Values are the means ± SEM; *n* = 5, 3, and 5 mice in *Mdm1*^+/+^, 7 weeks *Mdm1*^−/−^ mice, and 18 weeks *Mdm1*^−/−^ mice, respectively (*P < 0.05, ***P < 0.001 by one-way ANOVA). **d** Scheme of retinal degeneration induced by *Mdm1* deficiency. OS, outer segment; ONL, outer nuclear layer; OPL, outer plexiform layer; INL, inner nuclear layer; CC, connecting cilium; BB, basal body; IS, inner segment; N, nucleus.

RPE cells, as professional phagocytes, play important roles in the maintenance of retinal structure and function [16]. Photoreceptor cells continuously generate new discs of OS from their base while RPE cells simultaneously phagocytose OS, maintaining a roughly constant length of photoreceptor cells [16]. Therefore, we investigated the role of RPE cells in retinal degeneration of *Mdm1*^−/−^ mice by immunofluorescence staining with Ezrin, an apical marker of the RPE cells due to its high abundance in apical microvilli [17]. In 53-week-old *Mdm1*^+/+^ mouse retinas, apical microvilli of RPE intercalated the outside of OS (Fig. 6a). In the *Mdm1*^−/−^ mice, the RPE microvilli extended to the OS and surrounded some nuclei of the ONL at 17 weeks of age. RPE microvilli surrounded the ONL in 35-week-old mice with loss of OS and IS and the INL in 53-week-old mice with loss of ONL (Fig. 6a). Through electron microscopy, we confirmed the invasion of RPE microvilli into the nuclei of the ONL at 18 weeks and the entrapment of the nuclei in the ONL by RPE microvilli at 36 weeks (Fig. 6b). By performing a TUNEL assay and immunofluorescence staining with cleaved caspase-3, we sought to determine whether the RPE microvilli entrapment of the nuclei in the ONL induced apoptotic cell death. As expected, the number of TUNEL-positive cells and cleaved caspase-3 positive cells increased with age in the ONL of the *Mdm1*^−/−^ mice (Fig. 6c and Supplementary Fig. S4a). Apoptotic nuclei in the ONL of the *Mdm1*^−/−^ mice were also observed by electron microscopy (Supplementary Fig. S4b). We also confirmed whether non-apoptotic cell death occurred by measuring the accumulation of cyclic guanosine monophosphate (cGMP) and poly-ADP-ribose polymerase (PARP) activity. As a result, cGMP accumulation was not observed in ONL of *Mdm1*^−/−^ mice, and PARP stained cells were observed only in ONL of 53-week-old *Mdm1*^−/−^ mice (Supplementary Fig. S4c and S4d). These results suggest that *Mdm1* ablation increased the apoptosis rate in the ONL, which might have been mediated by the entrapment of ONL nuclei by RPE microvilli. Taken together, our results suggest that *Mdm1* deficiency causes defects in specific IFT, resulting in an imbalance between continuous replenishment of OS discs via the CC in photoreceptor cells and the simultaneous phagocytosis of the OS by RPE cells, leading to apoptosis in the ONL and causing retinal degeneration (Fig. 6d).

## Discussion

In this study, we demonstrated that Mdm1 is localized between the basal body and the axoneme of cilia in various tissues, and at the CC of photoreceptor cells in the retina and that *Mdm1* ablation results in retinal degeneration due to defective IFT in photoreceptor cells in mice.

The Mdm1 protein has been reported to be in the photoreceptor sensory cilia proteome in humans [7] and to be localized in the centriole lumen of centrosomes in dividing cells and multiciliated cells [6]. *Mdm1* transcripts were previously detected in the ONL, INL and ganglion cell layers of the mouse retina [8]. In the present study, we demonstrated that the Mdm1 protein is localized not only at the centrosome but also between the basal body and the axonemes of cilia in various cells, and at the CC of photoreceptor cells in the retina, as determined by immunofluorescence staining and electron microscopy. In particular, the Mdm1 protein is not observed at the centrioles of the basal body in the cilia of various cells, suggesting differential roles of Mdm1 in centrosomes and cilia. To the best of our knowledge, this is the first report showing the precise localization of the Mdm1 protein in ciliated cells.

The association of *Mdm1* with retinal degeneration was previously reported in *arrd2* mice, which was identified by a positional cloning approach [8]. *Arrd2* mice were found to harbor a truncated form of the Mdm1 protein with 398 amino acids caused by a nonsense mutation in the *Mdm1* gene [8]. Although *arrd2* mice showed normal fundi, electroretinograms, and retinal histology at 6 months of age, these mice showed vessel attenuation, severe hypo- or hyperpigmentation, alterations in the RPE, the presence of retinal dots at 14 months of age, and complete loss of photoreceptor cells by 22 months [8]. The ERG response also showed progressive amplitude loss from 8 to 16 months and was completely lost at 22 months [8]. A histological analysis of the retina of *arrd2* mice confirmed a slight decrease in OS and ONL up to 9 months of age and complete loss of OS and ONL at 19 months [8]. Electron microscopy of *arrd2* mice revealed OS fragmentation at 9 months [8]. Consistent with this previous report, our results showed that *Mdm1*^−/−^ mice exhibited retinal degeneration with complete loss of IS, OS, ONL, and OPL, alterations in the RPE and pigmentary abnormalities, and loss of ERG response. In addition, diverse phenotypes of retinal degeneration in *Mdm1*^−/−^ mice appeared much earlier than in *arrd2* mice, with a more rapid progression: OS fragmentation was observed 7 weeks in the *Mdm1*^−/−^ mice vs. 9 months in the *arrd2* mice; complete loss of ONL and ERG response was observed at 35 weeks in the *Mdm1*^−/−^ mice vs. 22 months in the *arrd2* mice. While *arrd2* mice may harbor a truncated form of Mdm1 protein containing the 398 N-terminal amino acids of the 708 amino acids in the full-length protein, *Mdm1*^−/−^ mice express a much shorter form containing only the 76 N-terminal amino acids. Therefore, the functional defects of the mutant Mdm1 protein might be greater in the *Mdm1*^−/−^ mice than in the *arrd2* mice, leading to much earlier and more rapid retinal degeneration in the *Mdm1*^−/−^ mice.

In ERG of *arrd2* mice, the rod response was minimal at 16 months with some sparing of the cone responses [8]. This result suggests that retinal degeneration starts from rods. In our study with mice of earlier age than *arrd2* mice, compared to the scotopic condition, the photopic condition showed relatively less amplitude degradation (data not shown). Therefore, it is highly likely that the rod will be damaged first. Furthermore, GNAT2 and arrestin immunofluorescence staining showed that the number of cone soma in ONL of 7-week-old *Mdm1*^−/−^ mice was similar to that of *Mdm*^+/+^ mice. These data suggest that the thinning of ONL is primarily caused by a loss of rods in *Mdm1*^−/−^ mice.

IFT is critical for the development and maintenance of the OS of photoreceptor cells [18]. Photoreceptor OS undergoes rapid and continuous turnover with approximately 10% of the OS shedding daily from the distal tip because of phagocytosis by adjacent RPE cells [19]. Therefore, new proteins or photosensitive discs synthesized in the IS must be supplied to the OS via the CC, for which an efficient trafficking system is required [20]. A variety of genes involved in IFT, such as *Ift88* [21], *Ift122* [22], *Ift81* [23], *Ift172* [24], and *Ift52* [25], have been reported to be associated with retinal degeneration. In addition, Bardet–Biedl syndrome (BBS) is an autosomal recessive, genetically heterogeneous, pleiotropic ciliopathy characterized by retinal degeneration, cognitive impairment, polydactyly, obesity, hypogenitalism, and renal abnormalities [26]. Some components of the BBSome, a protein complex that mediates ciliary trafficking, are also related to retinal degeneration [27]. *Bbs4*^−/−^ mice exhibit defects in the transport of rhodopsin from the IS to the OS and showed a decreased OS area with a normal CC structure [28]. The histological and electron microscopic features manifesting in retinas of the *Mdm1*^−/−^ mice were similar to those of the *Ift88*^−/−^ [21], the *Bbs4*^−/−^ [28], and *Bbs8*^−/−^ mice [26]. In the retinas of these mice, loss of OS, IS, and ONL occurred, and disc disorganization was observed under electron microscopy. However, the mislocalized proteins in each null mice were different. Bbs4^−/−^ mice exhibit impaired translocation of transducin and arrestin as well as transport defects of rhodopsin and cone opsin [28]. In Bbs8^−/−^ mice, rhodopsin distribution was normal, but there was aberrant accumulation of vesicle fusion protein syntaxin-3, STX-3 [26].

Our findings that the Mdm1 protein localizes to the CC of photoreceptor cells and that *Mdm1* ablation did not alter the structure of the CC suggest that Mdm1 might be involved in IFT in photoreceptor cells. Indeed, *Mdm1*^−/−^ mouse retina exhibited increased immunofluorescence staining of opsins, a membrane protein in photosensitive discs, in the IS and ONL, resulting in accumulation of vesicles in the IS. It has been reported that rhodopsin, S-opsin, and M/L-opsin are all transported by IFT [15, 29, 30]. However, there was no difference in the distribution of GNAT2 and arrestin in *Mdm1*^−/−^ mice compared to *Mdm1*^+/+^ mice. Therefore, these results support the hypothesis that Mdm1 ablation causes specific IFT defects in both rods and cones.

We also demonstrated that defective IFT induced by *Mdm1* ablation resulted in mislocalization of opsins, and led to the apoptosis of photoreceptor cells mediated by RPE cells. The survival of photoreceptor cells depends on the proper maintenance of opsin polarity [31]. Defects in opsin localization have been reported to be associated with several causes of retinal degeneration, including human retinitis pigmentosa and retinal detachment. Although opsin mislocalization itself was reported to induce the apoptosis of rod cells through G protein activation of adenylate cyclase [31], imbalance between insufficient continuous supplements of OS from photoreceptor cells and simultaneous phagocytosis of the OS by RPE cells might contribute to retinal degeneration upon *Mdm1* deficiency.

Interactions between photoreceptor cells, generating new discs of the OS critical for capturing and processing visual stimuli, and RPE cells, phagocytosing OS, play important roles in retinal homeostasis as well as the pathogenesis of retinal degeneration, especially age-related macular degeneration (AMD) [19]. We demonstrated that while RPE microvilli are mainly present in the OS of the *Mdm1*^+/+^ mouse retina, they protrude into the IS and ONL and even surround some nuclei of the ONL in the *Mdm1*^−/−^ mouse retina, resulting in apoptosis of photoreceptor cells. In the naturally occurring retinal degeneration (*rd1*) mice, two caspase-independent apoptotic pathways have been reported [32]. Caspase-12, localized to the ER translocate to the nucleus in a cleaved activated form, and apoptosis-inducing factor (AIF) present in the mitochondria also translocate to the nucleus to execute DNA fragmentation. AIF plays an important role in apoptotic events and caspase-12 has a potentiating effect. We confirmed ONL apoptosis in *Mdm1*^−/−^ mice by TUNEL assay and immunofluorescence staining of cleaved caspase-3. TUNEL-positive cells were more numerous than cleaved caspase-3 positive cells in the *Mdm1*^−/−^ mouse retina, suggesting that caspase-independent AIF caused DNA fragmentation as in *rd1* mice.

Previous studies have shown that non-apoptotic cell death as well as apoptosis act as important cell death mechanisms in various rodent photoreceptor degeneration models. In this alternative cell death mechanism, elevated levels of cGMP were associated with an increase in calpain-type proteases or PARP activity [33]. We checked an alternative cell death mechanism in *Mdm1*^−/−^ mice, but there was no significant change in cGMP level and PARP activity. These results indicate that cell death in retinal degeneration of *Mdm1*^−/−^ mice is predominantly apoptotic. Our findings suggest that the balance between continuous OS supplements from photoreceptor cells and simultaneous OS phagocytosis by RPE cells is critical to the maintenance of the function and structure of photoreceptor cells and is linked to retinal degeneration due to *Mdm1* ablation.

Previous study reported that C57BL/6N mice have the *rd8* mutation, and although there are differences between vendors, fundus lesions and histologic retinal lesions may develop as early as 6 weeks of age in C57BL/6N mice [34]. We initially generated *Mdm1*^−/−^ mice from C57BL/6N mice by the CRISPR/Cas9 system and then crossed them with C57BL/6N wild-type mice. We obtained *Mdm1*^*+/+*^ or *Mdm1*^*−/−*^ mouse by mating male and female *Mdm1*^*+/−*^ mice and analyzed retinal degeneration between them. We could not observe retinal degeneration in *Mdm1*^*+/+*^ mice up to 80 weeks of age. Therefore, we concluded that *Mdm1* ablation causes retinal degeneration. In order to completely exclude the effect of ocular lesion caused by *rd8* mutation and to elucidate the function of the *Mdm1* gene, it is necessary to investigate *Mdm1*^*−/−*^ mice in the C57BL/6J background mice.

We tried to identify other phenotypes in *Mdm1*^−/−^ mice since defects in ciliary components induce ciliopathy with prevalent phenotypes, including polycystic kidney disease, retinal degeneration, obesity, skeletal malformation, and brain anomalies [35]. We performed rotation sensory and olfactory tests as well as basic phenotype analysis (data not shown). However, we did not find phenotypes in addition to retinal degeneration in the *Mdm1*^−/−^ mice. The reason that *Mdm1*^−/−^ mice exhibit only retinal degeneration might be due to the unique mechanism for maintaining the retinal structure and function, which requires continuous supplements of OS from photoreceptor cells via IFT and simultaneous phagocytosis of the OS by RPE cells. Further in-depth study is necessary to elucidate the precise role of Mdm1 in the IFT of photoreceptor cells and to analyze Mdm1 function in cilia of other cells and other possible phenotypes caused by *Mdm1* ablation.

Retinal degeneration in *Mdm1*^−/−^ mice exhibits some similarities to human AMD and progressive retinal dystrophy, including retinitis pigmentosa and cone-rod dystrophy [36]. However, no association of *MDM1* and human AMD has been reported, as determined by analysis of the database of Genome-Wide Association Study (dbGaP), specifically a set of participants in the age-related eye disease study (AREDS) cohort [8]. In addition, no evidence has been shown for the association of *MDM1* with human retinal dystrophy [37, 38]. Although *MDM1* may not be directly associated with susceptibility to AMD and retinal dystrophy, it may still play a role in the maintenance of retinal function and structure. Although the human macular OS is about twice the length of the mouse central retinal OS, the rate of regeneration is comparable. Thus, the phagocytic load in the human macula is approximately twice that of the mouse retina. However, since the photoreceptor density in mice is 3–4 times higher than in humans, the load on RPE is twice that of humans. In addition, the size of the RPE is also larger in mice than in humans. Thus, the phagocytic load per RPE in mice is more than three times that of humans [39]. Therefore, it is thought that retinal degeneration may occur faster and more severe in mice than in humans in situations such as defective IFT caused by Mdm1 deficiency.

In conclusion, the present study revealed a novel important function of *Mdm1* as a potent factor involved in specific IFT in photoreceptor cells, which contributes to the maintenance of retinal structure and function. Hence, *Mdm1* ablation results in retinal degeneration in mice.

## Materials and methods

### Antibodies

The following antibodies were used: anti-Mdm1 (17575-1-AP) (Proteintech, Rosemont, IL, USA); anti-γ-tubulin (sc-17787) (Santa Cruz Biotechnology, Dallas, TX, USA); anti-Ezrin (ab4069) (Abcam, Cambridge, UK); anti-acetylated α-tubulin (T6793), anti-Opsin (O4886) (Merck KGaA, Darmstadt, Germany); anti-S-opsin (AB5407), anti-M/L-opsin (AB5405), anti-cone arrestin (AB15282) (Sigma-Aldrich, Burlington, MA, USA); anti-GNAT2 (GTX134342) (GeneTex, Irvine, CA, USA); cleaved caspase-3 (9664) (Cell Signaling Technology, Danvers, MA, USA), cGMP (bs-3892R) (Bioss antibodies, Woburn, MA, USA).

### Animals

*Mdm1*^−/−^ mice, generated by CRISPR/Cas9 technology, were obtained from the Korea Mouse Phenotype Center (Seoul, Republic of Korea). *Mdm1*^+/+^, *Mdm*^+/−^, and *Mdm1*^−/−^ mice were maintained at a controlled room temperature with 50% humidity and a 12-h light-dark cycle. Animal experiments were performed in accordance with the guidelines of the Institutional Animal Care and Use Committee of Yeungnam University (YUMC-AEC2020-027).

### Genotyping

Mdm1 genotypes were determined by PCR of genomic DNA isolated from mouse tails using a HelixAmp Direct PCR kit (NanoHelix, Daejeon, Republic of Korea) with the following primers: F, TTGACCATGCGCGTTGGTAG, R1, TGGGACAAGCGTATCCTTCTTTCT, and R2, TGGGACAAGCGTATCCTTCTGA. Reactions initially were denatured at 94 °C for 5 min followed by 35 cycles at 94 °C for 15 s, 61.5 °C for 15 s, 72 °C for 15 s, and a final extension at 72 °C for 7 min.

### Cell culture

hTERT retinal pigment epithelium cells (hRPE) (ATCC, Manassas, VA, USA) were maintained in DMEM/F12 with 10% FBS, 50 units/ml penicillin, and 50 mg/ml streptomycin (WELGENE, Gyeongsan, Republic of Korea).

### Preparation of mouse embryonic fibroblasts (MEFs)

Male and female *Mdm*^+/−^ mice were mated, and MEFs were isolated from E13.5 embryos [40]. Following the female mice were euthanized by cervical dislocation, the uterus was separated, immersed in PBS, and cut open to separate the embryos. The embryos were removed from the embryonic sacs and transferred to a 60-mm culture dish. A part of the tail of the embryo was reserved for genotyping. Each embryo was chopped with a sterilized blade and dissociated by treatment with 2 ml of trypsin-EDTA for 15 min in a CO_2_ incubator. After inactivation of the trypsin, the embryo was transferred to a 150-mm culture dish and incubated in a CO_2_ incubator for 4 h. After replacing the medium, MEFs were maintained and analyzed.

### Reverse transcription-polymerase chain reaction (RT-PCR)

Total RNA was isolated from MEFs using TRIzol solution (Bioscience Technology, Daegu, Republic of Korea). RT-PCR was performed using MMLV reverse transcriptase (Promega Corp., Madison, WI, USA), 2.5 μM oligo-dT primers, and 1 mM dNTPs. The resulting cDNA was amplified by PCR using Super-Therm Taq DNA polymerase (SR Product, Kent, UK) and the following primers: *Mdm1* forward, AGGGGCTGAGTGAATACCAGA, *Mdm1* WT reverse, GGACAAGCGTATCCTTCTTTCTG, *Mdm1* KO reverse, GGGACAAGCGTATCCTTCTGA, *Gapdh* forward, ACCACAGTCCATGCCATCAC, *Gapdh* reverse, TCCACCACCCTGTTGCTGTA. PCR products were resolved on 2% agarose gels and visualized using SYBR Green I (Invitrogen, Waltham, MA, USA) with a FUSION Solo S UV transilluminator (Vilber, Collégien, France).

### Protein extraction

Testis tissues and retinas isolated from the eyes were homogenized for 20 s in ice-cold RIPA buffer (25 mM Tris–HCl, pH 7.6; 150 mM NaCl; 1% Triton X-100; 0.5% sodium deoxycholate; 0.1% SDS; 1 mM Na_3_VO_4_; and 5 mM NaF) with 1 mM phenylmethylsulfonyl fluoride using a FastPrep-24 bead homogenizer (MP Biomedicals LLC, Solon, OH, USA) and centrifuged at 13,200 rpm for 20 min at 4 °C. The protein concentration of the supernatant was quantified using a Pierce BCA protein assay kit (Thermo Fisher Scientific, Rockford, IL, USA).

### Western blot analysis

Proteins were separated on a 7% SDS polyacrylamide gel and subsequently transferred to nitrocellulose membranes (GE Healthcare Bio Sciences, Marlborough, USA). Membranes were incubated with TTBS buffer (20 mM Tris–HCl, pH 7.6; 150 mM NaCl; and 0.1% Tween-20) containing 5% skim milk for 30 min at room temperature followed by incubation overnight at 4 °C with a primary antibody against Mdm1. After washing with TTBS buffer three times for 10 min each time, the membranes were incubated with a secondary antibody conjugated with horseradish peroxidase (Cell Signaling Technology, Danvers, MA, USA) for 2 h at 4 °C. Antigen-antibody complexes were detected with luminol (Elpis-Biotech, Daejeon, Korea) and visualized using an ImageQuant LAS 4000 system (GE Healthcare Bio Sciences, Chicago, IL, USA).

### Basic phenotype analysis

Basic phenotype analysis between *Mdm1*^*+/+*^ and *Mdm1*^*−/−*^ mice (each, *n* = 8) up to 1-year-old was conducted at the Korea Research Institute of Bioscience and Biotechnology (Daejeon, Republic of Korea). Briefly, the following results were assessed: growth curves; open field, grip strength, SHIRPA and dysmorphology, acoustic startle and prepulse inhibition, fear conditioning, and intraperitoneal glucose tolerance tests; and blood insulin level, auditory brain stem response, body composition by dual-energy absorptiometry (DEXA), hematology and clinical blood chemistry, immunophenotyping with splenocytes, gross pathology, and organ weight measurements.

### Tissue preparation

Mice were anesthetized by intraperitoneal injection of avertin (0.25 mg/g body weight). After cardiac perfusion with ice-cold PBS, mouse eyeballs, testes, brains, and noses were carefully removed. Tissues were fixed in 10% buffered formalin for paraffin slide preparation or fixed in 2.5% glutaraldehyde at 4 °C for transmission electron microscopy. The nose was decalcified overnight in 5% EDTA.

### Immunofluorescence staining

hRPEs on slide glasses were fixed with 4% formaldehyde for 15 min and permeabilized with methanol for 10 min at 4 °C. Paraffin-embedded tissues were cut into 10-μm sections, deparaffinized, rehydrated, and boiled in sodium citrate buffer (10 mM sodium citrate and 0.05% Tween-20 at pH 6.0) for 10 min at 121 °C for antigen retrieval. Cells and tissue sections were blocked in blocking solution (5% bovine serum albumin and 0.3% Triton X-100 in PBS) for 1 h at room temperature and incubated with primary antibodies in blocking solution overnight at 4 °C. After washing three times with PBS for 5 min each time, tissue sections were incubated with fluorescently labeled secondary antibodies (1:200) (Alexa Fluor® 488 and 546, Thermo Fisher Scientific, Waltham, MA, USA) in blocking solution for 1 h at room temperature. Nuclei were stained with 1 μg/mL 4’,6-diamidino-2-phenylindole dihydrochloride (DAPI; Pierce Biotechnology, Rockford, IL, USA) for 5 min and tissue sections were mounted with fluorescence mounting medium (Dako, Glostrup, Denmark). Slides were visualized with a K1-Fluo confocal microscope (Nanoscope Systems, Daejeon, Republic of Korea) using a ×60 objective lens (Olympus, Tokyo, Japan) and recorded with and photographed with a K1-Imagine software (Nanoscope Systems, Daejeon, Republic of Korea).

### Terminal deoxynucleotidyl transferase-mediated dUTP nick end labeling (TUNEL) staining

The TUNEL assay was performed using an In Situ Cell Death Detection kit (Roche, Mannheim, Germany) according to the manufacturer’s protocol. Briefly, paraffin-embedded tissue sections were deparaffinized, rehydrated, and incubated in freshly prepared permeabilization solution (0.1% Triton X-100 and 0.1% sodium citrate) for 8 min. After washing with PBS, TUNEL reaction mixture was added to each section and incubated for 1 h at 37 °C in a humidified dark chamber. Following washing with PBS three times for 5 min, tissue sections were counterstained with 1 μg/mL 4’,6-diamidino-2-phenylindole dihydrochloride (DAPI; Pierce Biotechnology, Rockford, IL, USA) for 5 min. Tissue sections were mounted with fluorescence mounting medium (Dako, Glostrup, Denmark) and photographed under a confocal microscope. The total number of TUNEL-positive nuclei was counted in each retina.

### Poly-ADP-ribose polymerase (PARP) in situ activity assay

PARP assay was performed as previously described [33]. Briefly, unfixed cryosections cut into 5 μm were blocked with an avidin/biotin blocking kit (Vector Laboratories, Newark, CA, USA). After washing with PBS briefly, tissue sections were incubated in PARP reaction mixture (10 mM MgCl_2_, 1 mM DTT, 5 µM biotinylated NAD^+^ (Tocris, Bristol, UK) in 100 mM Tris buffer with 0.2% Triton X-100 (pH 8.0)) for 2 h at 37 °C. After washing 2 times with PBS for 5 min each time, biotin incorporation was detected by treating avidin-Alexa Fluor 488 conjugate at room temperature for 1 h.

### Silver-enhanced diaminobenzidine-sulfide and immunoelectron microscopy

Paraffin-embedded tissue sections (10 μm) were immunohistochemically stained with an anti-Mdm1 antibody (1:4000 in the retina or 1:2000 in other tissues) or anti-γ-tubulin antibody (1:200) using a Dako EnVision Detection Systems kit (Dako, Glostrup, Denmark). Immunoelectron microscopy was performed according to the silver-enhanced diaminobenzidine-sulfide method [41]. Briefly, tissue sections stained with 3,3’-diaminobenzidine were transferred to a container with 2% sodium acetate (pH 8.7). After washing with distilled water, the slides were incubated in 0.1% sodium sulfite (pH 7.0) for 5 min and washed with 2% sodium acetate (pH 8.7) four times for 5 min each time. Thereafter, the slides were incubated in silver developer solution for 5 min. Tissue sections were incubated in 1% acetic acid for 5 min, quickly rinsed in 2% sodium acetate, and incubated for 2 min in 0.05% gold chloride. After washing with 2% sodium acetate, the slides were incubated in 0.3% sodium thiosulfate for 5 min. Finally, the slides were transferred to container with PBS and processed for electron microscopy.

### Ultra-widefield fundus photography and fluorescence angiography

After anesthetizing the mice, the pupils were dilated, and five micrograms of sodium fluorescein (Alcon, Geneve, Switzerland) was administered intraperitoneally. In vivo scanning laser ophthalmoscopy fundus photography, fundus autofluorescence (FAF), and fluorescein angiography (FAG) at wide angles were performed using the Optos California system (Optos plc, Scotland, UK).

### Electroretinography (ERG)

In vivo electroretinography was performed using an HMsERG LAB System (OcuScience Inc., Nevada, USA). The mice were anesthetized and maintained on a temperature-controlled table. A drop of 2% hypromellose solution (Hycell, Samil Pharmaceuticals, Seoul, Republic of Korea) was placed on a rodent contact lens with a silver-embedded thread electrode to maintain contact with the cornea and to keep it moistened. The mice were placed under a 76-mm-diameter mini-Ganzfeld dome in darkness for uniform illumination of the eyes. The measurements were obtained according to the International Society for Clinical Electrophysiology of Vision (ISCEV) extended full-field ERG standards. The data were analyzed using ERGVIEW (OcuScience Inc., Henderson, NV, USA), and the combined rod and cone response values under scotopic conditions (3.0 cd∙s/m^2^) were analyzed.

### Transmission electron microscopy (TEM)

The eyes were fixed overnight in 2.5% glutaraldehyde at 4 °C, washed with 0.1 M phosphate buffer (pH 7.2), and postfixed with 2% osmium tetroxide for 90 min. After washing three times with 0.1 M phosphate buffer (pH 7.2) for 10 min each time, samples were dehydrated through a graded series of 50% to 100% ethanol and 100% propylene oxide and infiltrated in 1:1, 1:2, and 1:3 mixtures of propylene oxide:epon for 1 h. The samples were incubated in 100% EPON for 8 h and cured at 35 °C and 45 °C for 12 h at each temperature, followed by additional hardening at 60 °C for 48 h. After trimming, ultrathin (60 nm) sections were double stained with 2% uranyl acetate for 25 min and 1% lead citrate for 15 min. The sections were visualized at 75 kV with an H7000 transmission electron microscope (Hitachi, Tokyo, Japan).

### Statistical analysis

The data are shown as the means ± SEM. Paired Student’s *t*-test, linear regression, and one-way ANOVA with Tukey’s post hoc test were performed using GraphPad Prism 7 software (GraphPad Software, San Diego, CA, USA). *P < 0.05, **P < 0.01, ***P < 0.001.

## Supplementary information

Supplementary Figure legends

Supplementary Figure S1

Supplementary Figure S2

Supplementary Figure S3

Supplementary Figure S4

Supplementary Information statistics

Original western blot of Fig. 1e

aj-checklist

Related Manuscript File

## Data Availability

All data generated and analyzed during this study are included in this published article and Supplementary Information files. Additional datasets are available from the corresponding author upon reasonable request.
