# Supplementary material for: Mdm1 ablation results in retinal degeneration by specific intraflagellar transport defects of photoreceptor cells
Source: Cell Death Dis. 2022 Sep 28;13(9):833. doi: 10.1038/s41419-022-05237-2 (PMC9519634; doi:10.1038/s41419-022-05237-2)
Supplement: Supplementary file 1 — Supplementary Figure legends [file 41419_2022_5237_MOESM1_ESM.docx]

**Supplementary Information**

**Supplementary Figure legends**

**Supplementary Figure S1. Localization of Mdm1 protein in cultured cells and cells of various tissues in *Mdm1*^+/+^ and *Mdm1*^-/-^ mice. a.** Immunofluorescence staining with Mdm1 and γ-tubulin in hRPE, and *Mdm1*^+/+^ and *Mdm1*^-/-^ MEFs. Scale bar, 10 μm. Immunoelectron microscopic analysis of Mdm1 and γ-tubulin (**b-e**). **b.** Ependymal cells of the ventricle. **c.** Choroid plexus epithelial cells. **d.** Olfactory cells in the nose. **e.** Nasal epithelial cells. Scale bar, 100 nm.

**Supplementary Figure S2. TEM analyses of retinal structures in *Mdm1*^+/+^ and *Mdm1*^-/-^ mice. a.** TEM images of the retinas in male *Mdm1*^+/+^ or *Mdm1*^-/-^ mice at the indicated ages. Scale bar, 10 μm. **b**. Ultrastructure of the IS of photoreceptor cells. Arrows indicate longitudinal fragmentation of the IS. Scale bar, 1 μm. **c.** Autophagosomes in the IS of photoreceptor cells. Arrowheads indicate normal autophagosomes, while arrows indicate multilayered autophagic vesicles. Scale bar, 1 μm. **d.** TEM images of the retinas of P5 and P7 mice. Scale bar, 1 μm. **e.** TEM images of the retinas of P10 mice with either eyes opened or closed. Scale bar, 1 μm. RPE, retinal pigmented epithelial cell; OS, outer segment; CC, connecting cilium; IS, inner segment; ONL, outer nuclear layer; OPL, outer plexiform layer; INL, inner nuclear layer; IPL, inner plexiform layer; GCL, ganglion cell layer; and m, mitochondria.

**Supplementary Figure S3. TEM images of the CC and distribution of S-opsin, M/L- opsin, GNAT2, and cone arrestin in the photoreceptor cells of the retinas of *Mdm1*^+/+^ and *Mdm1*^-/-^ mice. a.** CC of photoreceptor cells in *Mdm1*^+/+^ and *Mdm1*^-/-^ mice. Scale bar, 1 μm. **b.** Immunofluorescence staining of S-opsin (red) or M/L-opsin (red) and γ-tubulin (green) in the photoreceptor cells of 7-week-old *Mdm1*^+/+^ and *Mdm1*^-/-^ mice. Scale bar, 10 μm. **c.** Immunofluorescence staining of GNAT2 (red) or cone arrestin (red) and γ-tubulin (green) in the photoreceptor cells of 7-week-old *Mdm1*^+/+^ and *Mdm1*^-/-^ mice. Scale bar, 10 μm. OS, outer segment; CC, connecting cilium; IS, inner segment; ONL, outer nuclear layer; INL, inner nuclear layer

**Supplementary Figure S4. TEM images of the ONL of photoreceptor cells and cell death analysis in *Mdm1*^+/+^ and *Mdm1*^-/-^ mice. a.** Immunofluorescence staining of cleaved caspase-3 (red) in the retinas of *Mdm1*^+/+^ or *Mdm1*^-/-^ mice at the indicated ages. Scale bar, 10 μm. **b.** Nuclei in the ONL in *Mdm1*^+/+^ and *Mdm1*^-/-^ mice. Arrows indicate the apoptotic nuclei observed in Mdm1^-/-^ mice. Scale bar, 5 μm. **c.** Immunofluorescence staining of cGMP (red) in the retinas of *Mdm1*^+/+^ or *Mdm1*^-/-^ mice at the indicated ages. Scale bar, 10 μm. **d.** PARP activity assay (green) in the retinas of *Mdm1*^+/+^ or *Mdm1*^-/-^ mice at the indicated ages. Scale bar, 10 μm. OS, outer segment; IS, inner segment; ONL, outer nuclear layer; OPL, outer plexiform layer; INL, inner nuclear layer
