# Supplementary figures and images for: Mdm1 ablation results in retinal degeneration by specific intraflagellar transport defects of photoreceptor cells
Source: Cell Death Dis. 2022 Sep 28;13(9):833. doi: 10.1038/s41419-022-05237-2 (PMC9519634; doi:10.1038/s41419-022-05237-2)

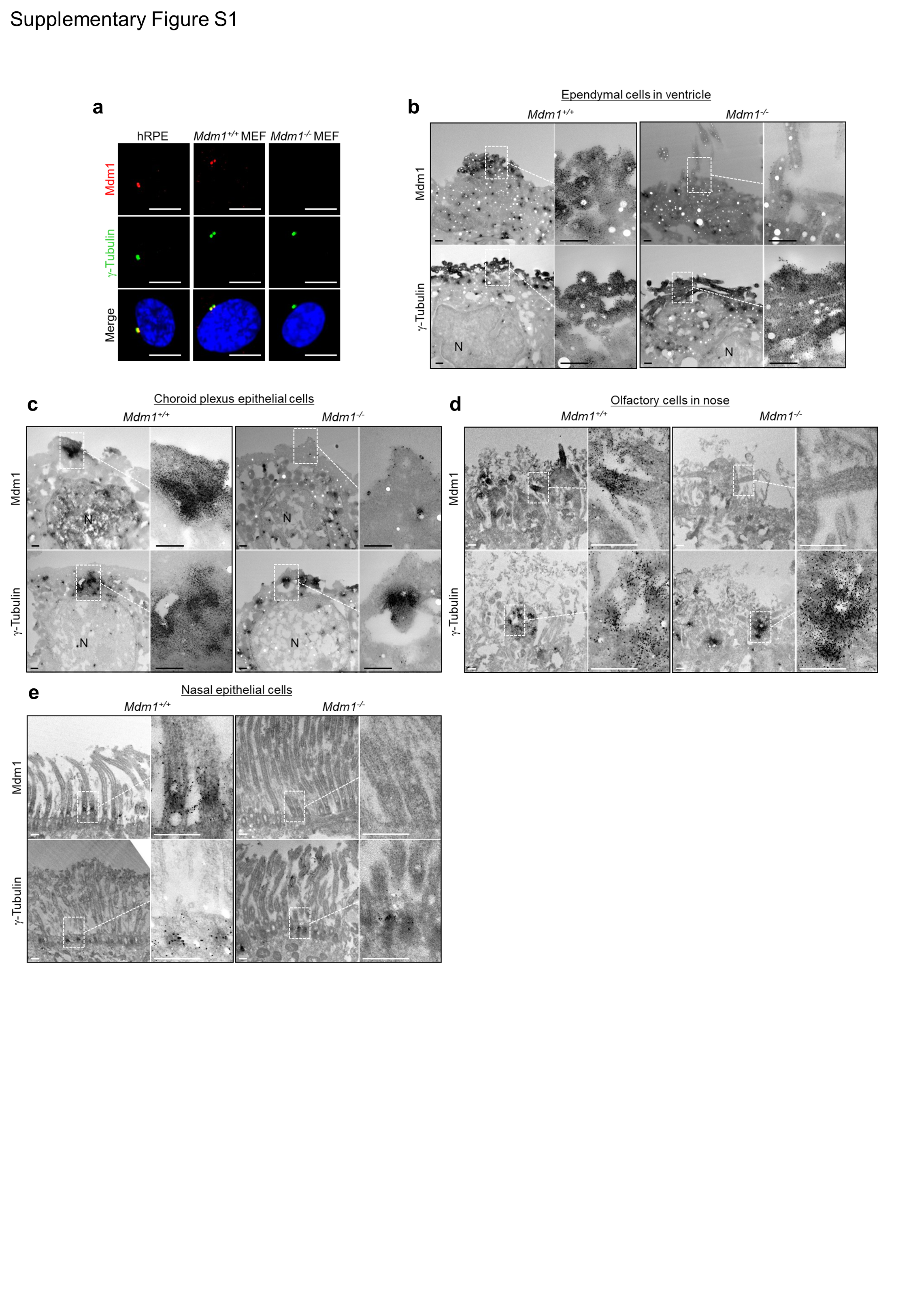

Supplement: Supplementary file 2 — Supplementary Figure S1 [file 41419_2022_5237_MOESM2_ESM.tif]

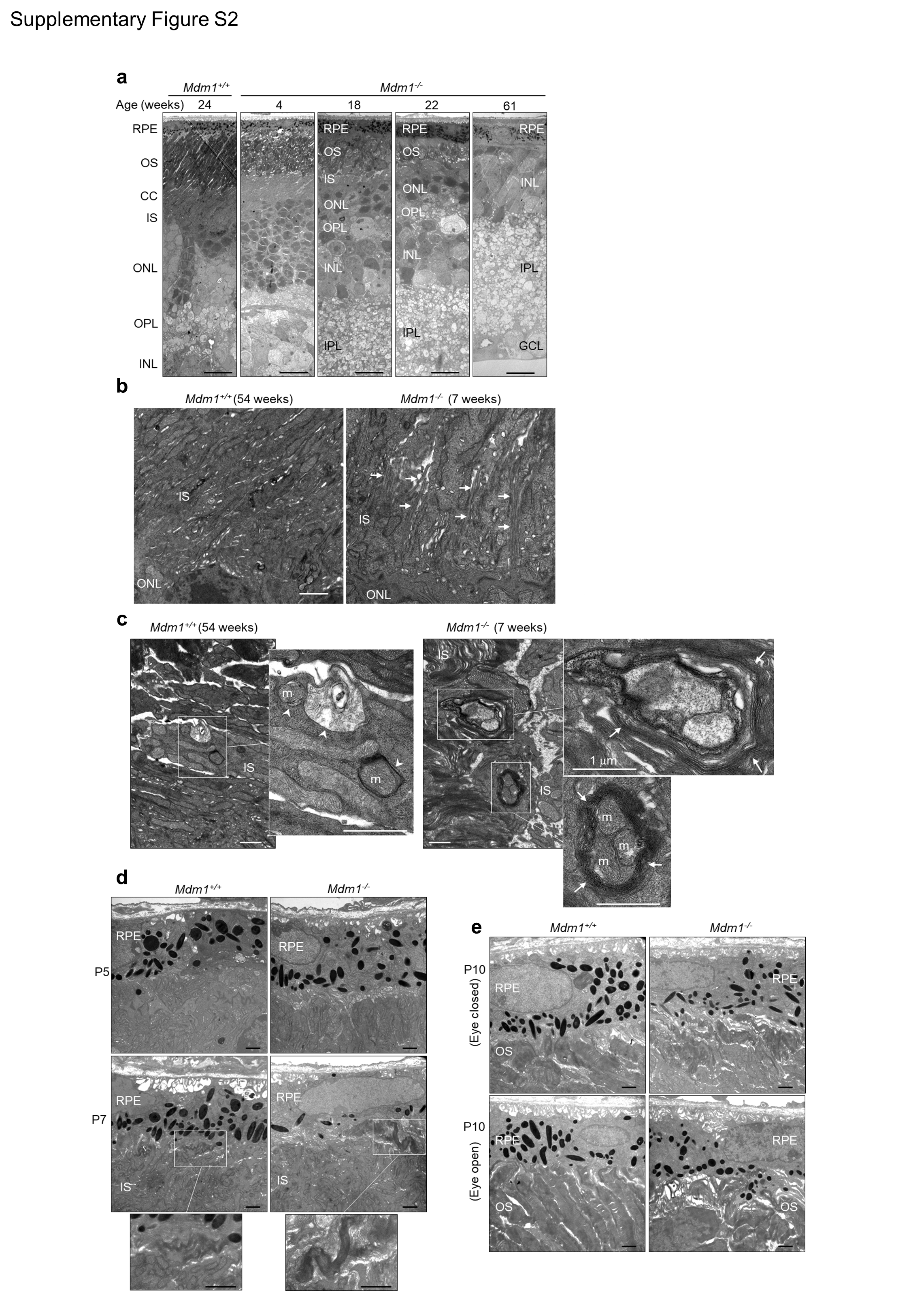

Supplement: Supplementary file 3 — Supplementary Figure S2 [file 41419_2022_5237_MOESM3_ESM.tif]

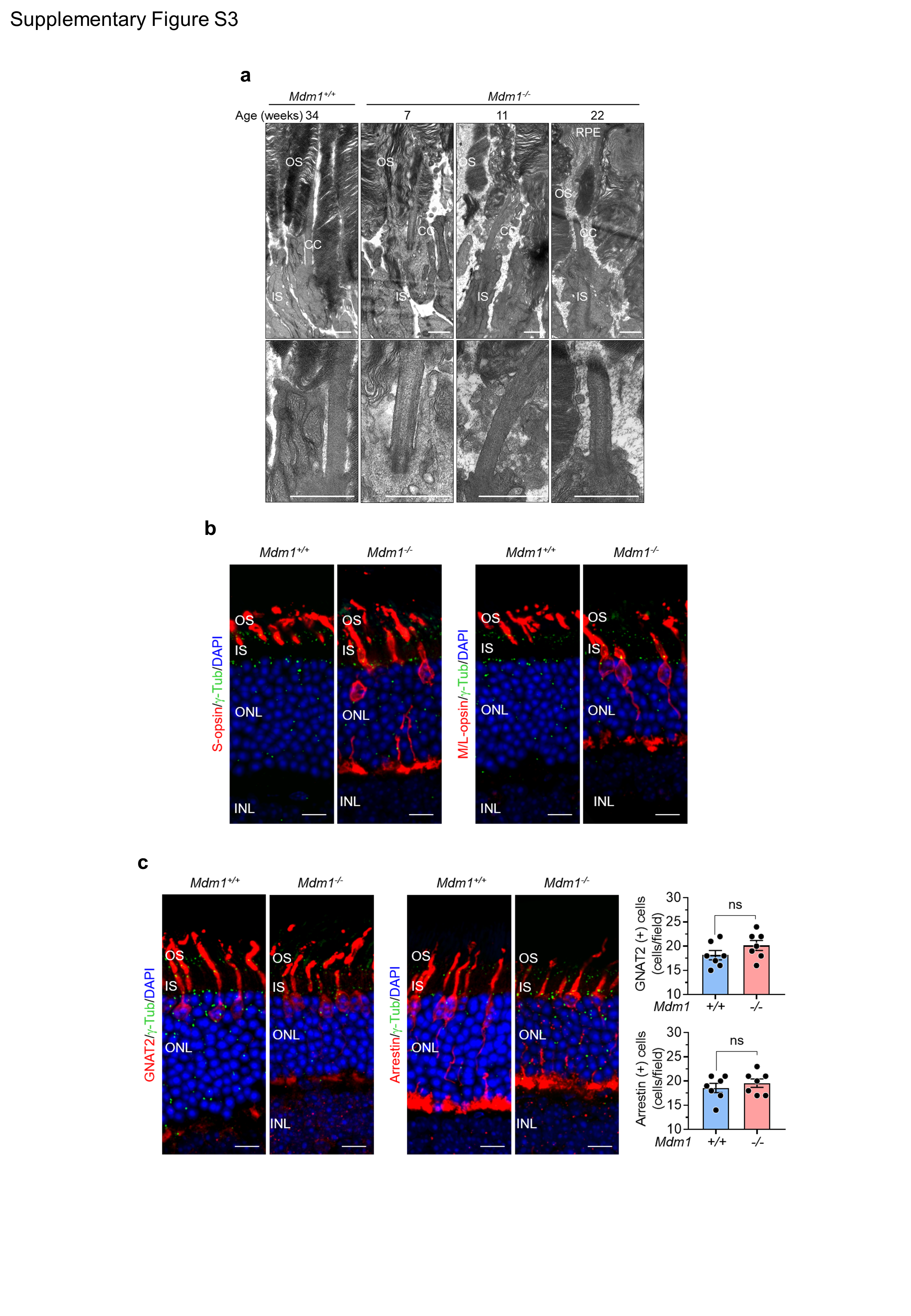

Supplement: Supplementary file 4 — Supplementary Figure S3 [file 41419_2022_5237_MOESM4_ESM.tif]

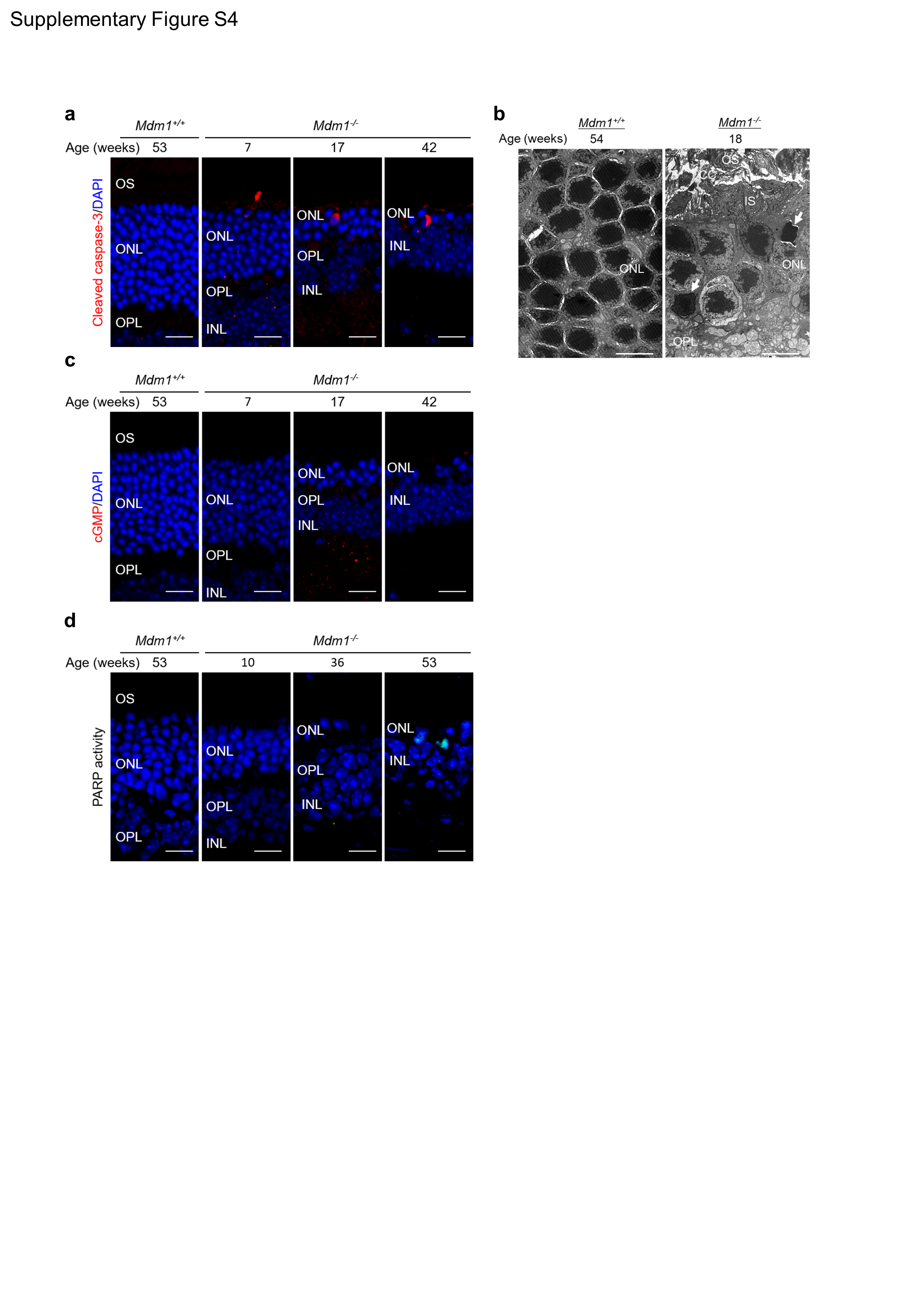

Supplement: Supplementary file 5 — Supplementary Figure S4 [file 41419_2022_5237_MOESM5_ESM.tif]
