# Supplementary material for: Mdm1 ablation results in retinal degeneration by specific intraflagellar transport defects of photoreceptor cells
Source: Cell Death Dis. 2022 Sep 28;13(9):833. doi: 10.1038/s41419-022-05237-2 (PMC9519634; doi:10.1038/s41419-022-05237-2)
Supplement: Supplementary file 9 — Related Manuscript File [file 41419_2022_5237_MOESM9_ESM.pdf]

DECLARATION OF CONTRIBUTIONS TO ARTICLE

ADMC

Manuscript Number: CDD-21-2281

Journal Name: *Cell Death & Differentiation* (the 'Journal')

Proposed Title of the Contribution:

Mdm1 ablation results in retinal degeneration by defective intraflagellar transport of photoreceptor cells (the 'Contribution)

Author(s):

Youlim Son, Soo-Jin Kim, Hwa-Young Kim, Junyeop Lee, Jae-Ryong Kim (the 'Authors')

For all *CDD* articles, each person named as an author in the published version must be able to show he or she has contributed substantially to the article.

Authorship credit should be based on 1) substantial contributions to conception and design, acquisition of data, or analysis and interpretation of data; 2) drafting the article or revising it critically for important intellectual content; and 3) final approval of the version to be published. Authors should meet conditions 1, 2 and 3.

Any person who cannot be shown to have made a substantial contribution to the article cannot be listed as an author in the final version. The name of any person who is deemed to have made a minor contribution can, however, appear in the Acknowledgments section of the article.

Please complete the table below to indicate the contributions of all named authors to the manuscript.

Author Full Name:	Specification of Contribution to the Manuscript:
Youlim Son	Y.S. designed and performed the experiments, analyzed the data, and wrote the manuscript
Soo-Jin Kim	S-J. K. performed ERG, analyzed the data, and wrote the manuscript
Hwa-Young Kim	H-Y. K. analyzed the data and reviewed the manuscript
Junyeop Lee	J.L conceived and designed the research project, arranged the data, and wrote the manuscript
Jae-Ryong Kim	J-R.K. conceived and designed the research project, arranged the data, and wrote the manuscript

Please complete the table below to indicate the contributions of all named authors to the figures.

Figure 1:

Fig. 1A: Youlim Son, Junyeop Lee, Jae-Ryong Kim
Fig. 1B: Youlim Son, Junyeop Lee, Jae-Ryong Kim
Fig. 1C: Youlim Son, Junyeop Lee, Jae-Ryong Kim
Fig. 1D: Youlim Son, Junyeop Lee, Jae-Ryong Kim
Fig. 1E: Youlim Son, Junyeop Lee, Jae-Ryong Kim

Figure 2:

Fig. 2A: Youlim Son, Hwa-Young Kim, Junyeop Lee, Jae-Ryong Kim
Fig. 2B: Youlim Son, Hwa-Young Kim, Junyeop Lee, Jae-Ryong Kim
Fig. 2C: Youlim Son, Hwa-Young Kim, Junyeop Lee, Jae-Ryong Kim
Fig. 2D: Youlim Son, Hwa-Young Kim, Junyeop Lee, Jae-Ryong Kim
Fig. 2E: Youlim Son, Hwa-Young Kim, Junyeop Lee, Jae-Ryong Kim
Fig. 2F: Youlim Son, Hwa-Young Kim, Junyeop Lee, Jae-Ryong Kim
Fig. 2G: Youlim Son, Hwa-Young Kim, Junyeop Lee, Jae-Ryong Kim
Fig. 2H: Youlim Son, Hwa-Young Kim, Junyeop Lee, Jae-Ryong Kim

Figure 3:

Fig. 3A: Soo-Jin Kim, Youlim Son, Hwa-Young Kim, Junyeop Lee, Jae-Ryong Kim
Fig. 3B: Soo-Jin Kim, Youlim Son, Hwa-Young Kim, Junyeop Lee, Jae-Ryong Kim
Fig. 3C: Soo-Jin Kim, Youlim Son, Hwa-Young Kim, Junyeop Lee, Jae-Ryong Kim
Fig. 3D: Soo-Jin Kim, Youlim Son, Hwa-Young Kim, Junyeop Lee, Jae-Ryong Kim

Figure 4:

Fig. 4A: Youlim Son, Junyeop Lee, Jae-Ryong Kim
Fig. 4B: Youlim Son, Junyeop Lee, Jae-Ryong Kim
Fig. 4C: Youlim Son, Junyeop Lee, Jae-Ryong Kim
Fig. 4D: Youlim Son, Junyeop Lee, Jae-Ryong Kim
Fig. 4E: Youlim Son, Junyeop Lee, Jae-Ryong Kim
Fig. 4F: Youlim Son, Junyeop Lee, Jae-Ryong Kim

Figure 5:

Fig. 5A: Youlim Son, Junyeop Lee, Jae-Ryong Kim
Fig. 5B: Youlim Son, Junyeop Lee, Jae-Ryong Kim

Figure 6:

Fig. 6A: Youlim Son, Junyeop Lee, Jae-Ryong Kim
Fig. 6B: Youlim Son, Junyeop Lee, Jae-Ryong Kim
Fig. 6C: Youlim Son, Junyeop Lee, Jae-Ryong Kim
Fig. 6D: Youlim Son, Junyeop Lee, Jae-Ryong Kim

Signed for and on behalf of the Author(s):	Print Name:	Date:
YOULIM SON	Youlim Son	6th Oct 21
